# COVID-19-Associated Mucormycosis: A Matter of Concern Amid the SARS-CoV-2 Pandemic

**DOI:** 10.3390/vaccines10081266

**Published:** 2022-08-06

**Authors:** Pankaj Chandley, Priyanka Subba, Soma Rohatgi

**Affiliations:** Department of Biosciences and Bioengineering, Indian Institute of Technology Roorkee, Roorkee 247667, India

**Keywords:** mucormycosis, COVID-19, immunotherapies, antibodies, cytokine therapy, combination therapy, black fungus, antifungal drugs

## Abstract

Mucormycosis is an invasive fungal infection caused by fungi belonging to order Mucorales. Recently, with the increase in COVID-19 infections, mucormycosis infections have become a matter of concern globally, because of the high morbidity and mortality rates associated with them. Due to the association of mucormycosis with COVID-19 disease, it has been termed COVID-19-associated mucormycosis (CAM). In the present review, we focus on mucormycosis incidence, pathophysiology, risk factors, immune dysfunction, interactions of Mucorales with endothelial cells, and the possible role of iron in Mucorales growth. We review the limitations associated with current diagnostic procedures and the requirement for more specific, cost-effective, convenient, and sensitive assays, such as PCR-based assays and monoclonal antibody-based assays for the effective diagnosis of mucormycosis. We discuss the current treatment options involving antifungal drug therapies, adjunctive therapy, surgical treatment, and their limitations. We also review the importance of nutraceuticals-based therapy for the prevention as well as treatment of mucormycosis. Our review also highlights the need to explore the potential of novel immunotherapeutics, which include antibody-based therapy, cytokine-based therapy, and combination/synergistic antifungal therapy, as treatment options for mucormycosis. In summary, this review provides a complete overview of COVID-19-associated mucormycosis, addressing the current research gaps and future developments required in the field.

## 1. Introduction

Fungal infections have been often neglected, despite their high morbidity and mortality rates worldwide [1]. Fungal diseases have a considerable impact on health on a global scale, which needs to be addressed by developing appropriate diagnostics and therapeutics for fungal infections [2]. Mucormycosis, also known as black fungus, is a rare but life-threatening, serious fungal infection caused by fungi belonging to the order Mucorales.

### 1.1. Overview of Mucormycosis

Mucormycosis is an angioinvasive fungal infection caused by fungal species belonging to different genera, which include *Rhizopus*, *Apophysomyces*, *Rhizomucor*, *Lichtheimia*, *Cunninghamella*, *Mucor*, and *Saksenaea* [3]. Globally, the most common causative agent of mucormycosis is *Rhizopus arrhizus* [4,5]. Other emerging *Rhizopus* species involved in causing mucormycosis infection are *R. homothallicus* and *R. microsporus* [6,7]. Various *Rhizopus* species that are involved in causing rhino-orbital-cerebral mucormycosis have been reported in abundance in the environment [8,9]. In addition to *Rhizopus*, *Apophysomyces* is considered as the second most common fungal Genus causing mucormycosis infection in Asia. The fungal species *A. variabilis*, which causes cutaneous mucormycosis, accounts for 60% of mucormycosis cases in India [5,10]. Various other species, including *Lichtheimia ramosa*, *Thamnostylum lucknowense*, and *Mucor irregularis*, have also been reported in India [11,12,13]. 

Many biotic environments, such as outdoor and indoor habitats, along with various food items, may contribute to the growth and persistence of Mucorales in a wide range of environmental conditions [14]. Soil is a major outdoor habitat for Mucorales. In particular, *A. elegans* has been reported to be present in soil from tropical climates [15]. *Mucorales* spp., particularly *Rhizopus* spp., require a high moisture content for growth and are considered as hydrophilic. Mucorales tend to settle down with liquid droplets present in the air, which could be a probable reason why Mucorales are not detected in the air samples obtained from indoor habitats [14]. A predominance of *Rhizopus*, *Rhizomucor*, and *Mucor* spp. has been reported in air-conditioning filters, which indicates the persistence of *Mucorales* spp. in settled dust particles [16]. A variety of *Mucorales* spp. (*Rhizopus*, *Lichtheimia*, and *Syncephalastrum*) has also been reported in food sources. As such, Mucorales-contaminated food items may also be a risk factor for acquiring mucormycosis in immunocompromised individuals [17].

**Figure 1:**
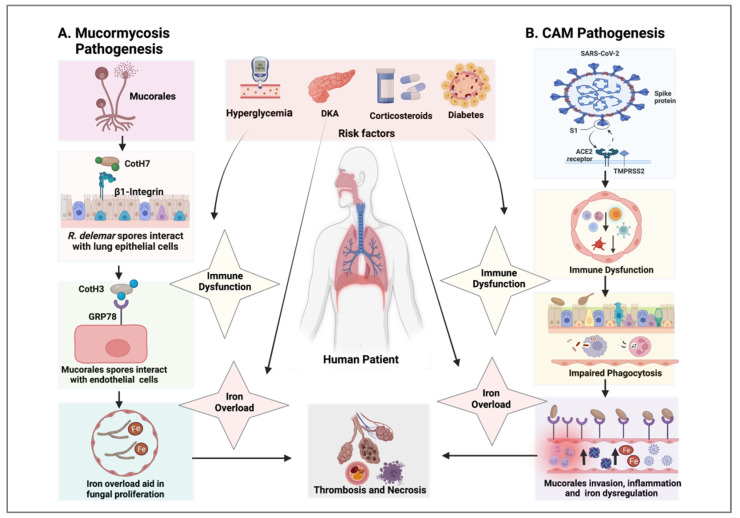
Panel A. Mucormycosis pathogenesis (in the absence of COVID-19): Mucorales sporulation produces spores that are inhaled through the respiratory system. Fungal spores interact with the lung epithelial surface through the CotH7 protein, which binds to the β-1 integrin receptor. This receptor–ligand interaction leads to epithelial cell invasion and damage. Mucorales also interacts with the glucose-regulated 78 kDa protein (GRP78) present on the surface of lung endothelial cells through its spore coat protein (CotH3), which results in cell invasion and tissue damage. Major risk factors are diabetes mellitus (DM), corticosteroid therapy, hyperglycemia, and diabetic ketoacidosis (DKA). Hyperglycemia leads to immune dysfunction, which is characterized by impaired activity of phagocytes, including functional chemotaxis/oxidative and non-oxidative killing mechanisms. Impaired fungal phagocytosis contributes to increased Mucorales endocytosis. DKA causes the iron overload in blood serum that contributes to Mucorales growth and proliferation, which ultimately results in blood vessel thrombosis via the occlusion of blood supply, cell damage, and tissue necrosis. Panel B. CAM pathogenesis (in the presence of COVID-19): The interaction of COVID-19 spike protein with host angiotensin-converting enzyme 2 (ACE2) and cell surface serine protease TMPRSS2 leads to downstream pathways, which results in virus entry and proliferation. Severe COVID-19 infection leads to immune dysfunction via the downregulation of B cells, T cells (lymphopenia), NK cells, neutrophils (neutrophil dysfunction), macrophages, and platelets (thrombocytopenia). COVID-19-mediated immune dysfunction can cause impaired phagocytosis of Mucorales spores, which results in Mucorales immune evasion. Mucorales spores find their way to the basement membrane where it adheres with laminin and collagen IV proteins of the extracellular matrix. Mucorales then interacts with endothelial cells through CotH3 protein via the GRP78 receptor, leading to Mucorales invasion. SARS-CoV-2 infection enhances GRP78 expression and also dysregulates iron homeostasis, thereby increasing the plasma concentration of free iron. Free iron is utilized by Mucorales for growth and proliferation, enhancing the extent of Mucorales invasion, cell damage, thrombosis, and tissue necrosis. Major risk factors include DM, corticosteroid therapy, DKA, and hyperglycemia. Created with BioRender.com.

Mucorales spores dispersed in dust particles gain entry into a human host via the respiratory tract and skin or a breach in mucosal barrier/traumatized sites, and fungal spore germination and proliferation lead to cutaneous necrotizing fasciitis and/or disseminated mucormycosis infections [15,18]. In immunocompetent hosts, patrolling phagocytes (macrophages and neutrophils) of the lung alveolar region are responsible for controlling the germination and proliferation of Mucorales spores by producing reactive oxygen species and anti-microbial peptides, such as defensins [19]. Furthermore, the presence of specialized iron-binding proteins, which sequester iron in serum and endothelial cells that regulate permeability, control mucormycosis. Mucormycosis infections are rare in immunocompetent hosts and typically occur in the setting of trauma. However, in immunocompromised hosts, these defense mechanisms break down. Mucorales multiply rapidly upon encountering a favorable environment in immunocompromised individuals. The impairment of host defense mechanisms, such as decreased phagocytosis during immunocompromising conditions, including hyperglycemia and glucocorticoid treatment, aids fungal growth and leads to disease progression. In diabetic ketoacidosis (DKA), the acidic pH of the serum causes the dissociation of free iron from sequestering proteins, and free iron contributes to the rapid growth and proliferation of pathogens. Mucorales can also downregulate host defense genes, which results in host immune evasion [20,21]. The interaction of Mucorales spores containing CotH7 with the β1-integrin receptor present in epithelial cells, followed by endothelial invasion via CotH3 and GRP78 (glucose-regulated protein) receptor interaction, is a crucial step in the pathogenesis of mucormycosis. Mucorales proliferation causes the occlusion of blood supply (thrombosis), which results in tissue necrosis and endothelial cell damage [22]. Endothelial cell damage leads to fungal angioinvasion and further dissemination. The pathogenesis of mucormycosis is described in Figure 1 (Panel A). 

Mucormycosis infection is mainly associated with diabetic and immunocompromised patients [23,24]. Other risk groups include patients with hematological malignancies, severe kidney or liver diseases, hyperglycemia, DKA, solid organ transplant, and immunological disorders [25,26]. Mucormycosis is a non-contagious disease. The symptoms of mucormycosis may vary in different patients. Some of the early symptoms include nasal pain, loss of vision, headache, fever, blackish nasal discharge, facial pain on one side, and swelling in the mouth [27]. The ignorance of early symptoms for a long period of time may lead to systemic dissemination to other organs. Mucormycosis affects many organs in the human body. The main organs affected in mucormycosis infections are the nose, sinuses, lungs, eyes, and brain [28]. On the basis of the multiple anatomical sites involved, mucormycosis is categorized further into six major types: pulmonary, cutaneous, disseminated, rhino-orbital, rhino-orbital-cerebral, and gastrointestinal. Various other miscellaneous types of mucormycosis affecting different organs can also be observed in humans [29,30].

*Mucorales* spp. are identified by a direct microscopy examination and fungal cell culture. Mucorales specimen are cultured on Sabouraud dextrose agar at a 30–37 °C temperature that forms grayish or black colonies [31]. Other diagnostics, such as histopathological examination with Hematoxylin and Eosin (H&E) staining, fungal DNA sequencing, and MALDI-TOF-based identification, can also be used for mucormycosis [32,33]. In rhino-cerebral mucormycosis, because of its detectable symptoms, it can be diagnosed early and managed by the surgical removal of infected tissue followed by antifungal therapy. However, in the cases of disseminated and pulmonary mucormycosis, due to the involvement of deep tissues, its diagnosis becomes difficult. Additionally, the lack of visible symptoms in disseminated and pulmonary mucormycosis makes early diagnosis challenging, which further impairs the management of mucormycosis infection [31]. Although the culture-based identification of Mucorales specimens is strongly recommended [31], this approach is a time-consuming and laborious process. For the rapid identification of the Mucorales species, MALDI-TOF is recommended [31]. Most laboratories in developing countries lack such expensive diagnostic tools; therefore, cost-effective diagnostics, such as PCR-based and antibody-based tests, need to be developed for mucormycosis. In order to treat mucormycosis infection, amphotericin B is considered as a standard treatment option [34,35]. During the un-availability of amphotericin B, isavuconazole, and posaconazole, itraconazole administration may be considered [36].

### 1.2. Overview of COVID-19-Associated Mucormycosis

During the recent SARS-CoV-2 pandemic, an increase in mucormycosis cases has been reported, causing severe infections in COVID-19 patients [37]. Mucormycosis cases associated with pre-existing COVID-19 disease are referred to as COVID-19-associated mucormycosis (CAM). Currently, the SARS-CoV-2 pandemic has resulted in a significant loss of health and economy all over the world. Increased incidences of COVID-19-associated diseases, such as CAM, further complicate COVID-19 disease management. Such invasive fungal infections are increasing at a rapid pace and are a matter of concern during the current pandemic. Uncontrolled co-morbidities, such as diabetes mellitus (DM), and the increased use of corticosteroids are major risk factors that may contribute to COVID-19-associated fungal infections, which may also increase the severity of COVID-19 infection [38,39]. Hyperglycemia, DM, DKA, and corticosteroid therapy are considered as risk factors for both CAM and severe COVID-19 infection, independently. The management of fungal infections is a challenging task in hospital settings. According to some reports, there is a high risk of contracting COVID-19-associated fungal infection in COVID-19 patients, due to the requirement of mechanical ventilation. People who need intensive care and prolonged hospitalization are the individuals most vulnerable to mucormycosis infection [32,33]. Severe COVID-19 patients undergoing treatment in intensive care units (ICUs) are the individuals at a greater risk of acquiring mucormycosis infection [40].

**Figure 2:**
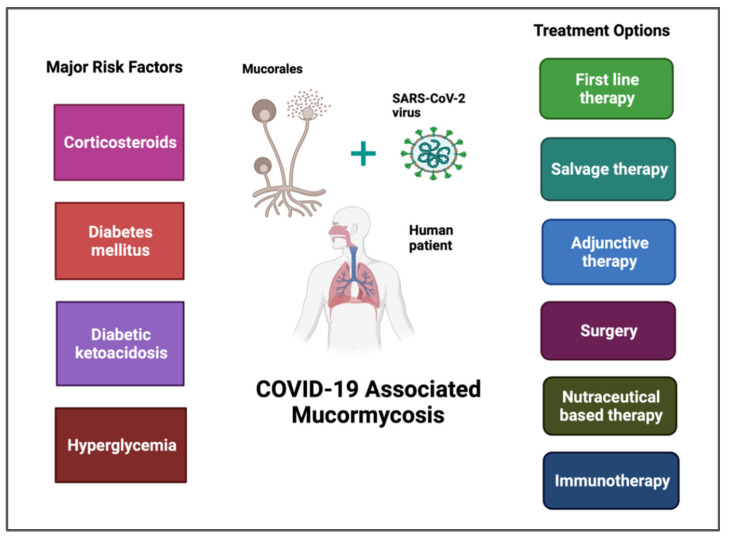
COVID-19-associated mucormycosis. Left Column: Major risk factors include corticosteroids, DM, DKA, and hyperglycemia. Right Column: Treatment options include first-line therapy (liposomal amphotericin B and amphotericin B deoxycholate), salvage therapy (isavuconazole, itraconazole, and posaconazole), adjunctive therapy (iron chelators (lactoferrin), saturated solution of potassium iodide, statins, and anti-inflammatory drugs (Aspirin)), surgery (debridement), nutraceuticals, and immunotherapy (IFN-γ, anti-CotH3 antibodies, IL-2 pre-stimulated NK cells, and G-CSF mobilized granulocyte transfusion). Created with BioRender.com.

The major risk factors of CAM include corticosteroid therapy, DM, DKA, and hyperglycemia, which are presented in Figure 2 (left column). Various factors involved in CAM pathogenesis, such as immune dysfunction, hyperferritinemia, iron overload, inflammation, thrombosis, and necrosis, are presented in Figure 1 (Panel B). CAM cases may be controlled by applying preventive measures (the maintenance of proper hygiene practices, regular decontamination of hospital equipment, and air-conditioning vents) and creating general awareness in public and medical settings, but the uncontrolled co-morbidities and underlying hematological malignancies are responsible for the majority of CAM-associated fatalities [41]. Recently, two real-time PCR assays, out of which one was developed in-house and another is available as a commercial kit (Mucorgenius™ by Pathonostics, the Netherlands), were introduced for the diagnosis of mucormycosis [42]. CAM-treatment options include: first-line therapy (liposomal amphotericin B and amphotericin B deoxycholate); salvage therapy (isavuconazole, itraconazole, and posaconazole); surgery (debridement); adjunctive therapy (iron chelators (lactoferrin), saturated solution of potassium iodide, statins, and anti-inflammatory drugs (Aspirin)); nutraceuticals; and immunotherapy (IFN-γ, anti-CotH3 antibodies, IL-2 pre-stimulated NK cells, and G-CSF mobilized granulocyte transfusion), which are presented in Figure 2 (right column).

Due to the immense burden of CAM, the identification of risk factors, understanding Mucorales pathophysiology, and development of effective diagnostics and therapeutics against mucormycosis is the need of the hour. In the current article, we review information obtained from various multicenter and meta-analysis studies, systematic reviews, narrative reviews, and recent research articles on CAM from various electronic platforms, such as PubMed, Google Scholar, Research Gate, and Scopus. We discuss several case reports of CAM based on various parameters, such as the number of patients, age, gender, associated co-morbidity, involvement of COVID-19, and treatment option used. Furthermore, this review also summarizes the current and updated information on the incidence, risk factors (host as well as environmental factors), pathophysiology and immune dysfunctions, role of iron metabolism in Mucorales growth, diagnostics, and possible treatment options for mucormycosis. 

## 2. Incidence of COVID-19-Associated Mucormycosis

According to few previous estimates, the prevalence of mucormycosis in the U.S. and Europe was 0.01–0.02 per 100,000 people [40]. However, a rise in CAM cases has been observed worldwide amid the SARS-CoV-2 pandemic. Both developed and developing countries are facing the continuous burden of extensive secondary fungal infections amid the SARS-CoV-2 pandemic. However, compared to developed nations, mucormycosis cases are more prevalent in developing countries and a majority of CAM cases (incidence rate: 0.14 per 1000 people) have been reported in India [43]. Notably, mucormycosis infections were very common (about 70 times higher than the global average) in India, even before the SARS-CoV-2 pandemic [44]. In a recent study, the symptoms of mucormycosis infection were reported within 7 days after the onset of COVID-19 infection in about 34.7% patients, while in the majority (64%) of the patients the mucormycosis symptoms appeared after 15 days of COVID-19 infection [45]. During the SARS-CoV-2 pandemic, various researchers reported an increase in mucormycosis cases globally, with DM being identified as the most common underlying condition [46,47,48,49,50,51,52,53,54,55,56,57,58,59,60,61,62,63,64,65,66,67,68,69,70,71,72,73,74,75]. A summary of various case reports is presented in Table 1. 

Recently, for estimating the global prevalence of CAM, Hussain et al. conducted a meta-analysis by pooling a sample size of 52,196 COVID-19 patients. They reported that CAM incidence was 50 times higher (7 per 1000 COVID-19 cases) than the previously available highest-incidence data (0.14 per 1000 population). A high mortality rate (26.9%) was found to be associated with CAM infection [76]. In another study, Nagalli et al. conducted a systematic review using electronically available data. They selected 115 COVID-19 patients with proved mucormycosis infection. They reported that 90% patients received steroids for COVID-19 treatment and DM was the most common (about 77.1%) co-morbidity for mucormycosis among them. Despite the presence of antifungal treatment, a high mortality rate (about 48.7%) was attributable to CAM infection [77]. More recently, a systematic review conducted by Kamat et al. reported rhino-orbital mucormycosis in the majority of CAM cases. More than 80% of cases were diabetic and showed a history of steroid administration. A mortality rate of 25.6% was reported, even after the administration of antifungal drug therapy [78].

From the above description, it can be concluded that both immunocompetent and immunocompromised patients were affected by mucormycosis infection [57,66,69]. DM [46,47,49,50,53,54,55,56,58,59,60,61,62,63,64,65,70,71,74,75], followed by corticosteroid therapy [50,51,52,56,58,59,60,61,63,64,65,70], DKA [46,55,56,67,75], and hyperglycemia [54,62,74] were reported as major risk factors contributing to CAM infection. Apart from pulmonary, rhino-orbital, rhino-cerebral, and disseminated mucormycosis, a variation in the anatomical locations of mucormycosis infection was observed. For instance, the involvement of rare locations, such as mandibular [73], sino-nasal [75], and paranasal [53,71] mucormycosis were reported. Several case reports (mentioned in Table 1) presented high mortality rates associated with CAM infection. 

It is evident from Table 1 that *Rhizopus* spp. is associated with the majority of CAM infections [47,48,49,54,62,63,67,68,69,74] followed by *Mucor* spp. [53,67,70,71] and *Lichtheimia* spp [46,49]. For COVID-19 diagnosis, an RT-PCR test is the most frequently used diagnostic test [46,47,48,49,50,51,52,53,55,57,58,61,62,63,64,65,66,67,69], followed by the COVID-19 antibody test [74], and cartridge-based nucleic acid amplification test (CBNAAT) [71]. Although standard amphotericin-B treatment followed by posaconazole, isavuconazole, and fluconazole treatment along with surgery improved the patient’s survival outcome, a high mortality rate (42.16%) was observed in CAM infections. Since both COVID-19 and mucormycosis infections are life-threatening, together they pose a considerable burden of mortality to mankind.

## 3. Risk Factors Associated with COVID-19-Associated Mucormycosis

The risk factors that contribute to mucormycosis are uncontrolled DM, uncontrolled use of corticosteroids, obesity, DKA, neutropenia, trauma, allogenic hematopoietic stem cell transplant, hematological malignancies, cytokine storm, and solid organ transplant [79]. Various studies conducted in India reported risk factors that may be associated with mucormycosis. For instance, Prakash et al. compared the incidence of DM-associated mucormycosis and found that north India contributed to 67% of cases, while only 22% of cases were reported in south India [58]. The percentages of cases reported in India with underlying disease as a risk factor for mucormycosis are hematological malignancies (1–9%), solid organ transplant (about 2.6–11%), and trauma (7.5–22%) [80,81,82,83,84]. Voriconazole treatment was reported as a risk factor for invasive fungal diseases [85,86]. Recently, Singh et al. reported the possible role of nasal microbiota imbalance as a contributing factor for the emergence of CAM [87]. Some risk factors that contribute to the majority of CAM cases are described below.

### 3.1. Corticosteroids

Corticosteroids are effective in the early management of COVID-19 infection [88,89]. However, COVID-19 patients having DM and treated with corticosteroid therapy are more likely to develop mucormycosis infection [60]. The excessive use of corticosteroids for more than three weeks may increase the risk of angioinvasive mucormycosis [90]. Corticosteroid therapy is considered as a risk factor for mucormycosis; however, in one study, a low dose of corticosteroids was found to be effective in 89% of ICU patients and also decreased the risk of mucormycosis. Additionally, a low dose of corticosteroids was found to be effective for good glycemic control, and as a result no mucormycosis was reported in 40% of diabetic patients [91]. A high dose of corticosteroid used in COVID-19 patients may affect the functioning of the host’s defense system and dysregulate glycemic control [92]. A thorough assessment is required before using a high dose of corticosteroids or immunosuppressants in COVID-19 patients, keeping in mind the underlying diseased condition and its associated risk of co-infection [93]. 

### 3.2. DM

DM is one of the factors contributing to mucormycosis infection. In a recent study, 57% of total mucormycosis patients had DM, and 18% of patients were diagnosed with DKA [58]. DM is also associated with the incidence of the rhino-orbital-cerebral type of mucormycosis [94]. DM acts as a major risk factor for mucormycosis that may elevate morbidity and mortality rates in COVID-19 patients [38]. SARS-CoV-2 infection causes a cytokine storm, which increases insulin resistance and also enhances the expression of ACE-2 receptors in pancreatic islets. In this way, the virus causes damage to pancreatic islets and leads to DKA [95]. Various studies reported DM as an underlying co-morbidity contributing to CAM infection [96,97]. 

Furthermore, a recently conducted study reported DM as the most common risk factor in 86.6% of mucormycosis-affected patients [45]. Uncontrolled DM in COVID-19 patients may be responsible for the convergence of COVID-19 and mucormycosis and increase in the severity of both infections. A sudden surge in mucormycosis cases was found to be associated with diabetic patients having poor glycemic control. In COVID-19 patients, glucocorticoid administration contributes to impaired glycemic control. Therefore, the use of glucocorticoids should be regulated to overcome the risk of CAM infection [98]. 

### 3.3. DKA

DKA is associated with autoimmune DM that may cause the destruction of the β cells of pancreatic islets and may lead to insulin deficiency [99]. Another study suggested that COVID-19 infection may impair the functioning of pancreatic islets (β cells) and lead to DKA in euglycemic patients [100]. COVID-19 patients may develop hyperglycemia, which indicates that damage is occurring in the pancreatic islet cells [101]. A systematic review reported that 77% of cases of type 2 DM patients had DKA [102]. 

### 3.4. Hyperglycemia

SARS-CoV-2 may cause insulin resistance and damage to β cells, which increases blood glucose level [103,104]. Hyperglycemia increases the availability of free iron, upregulates the expression levels of GRP78 and CotH3, and affects host defense responses that create favorable conditions for fungal growth and host endothelium invasion [26]. Additionally, hyperglycemia leads to immune dysfunction. It has been shown to impair the activity of phagocytes, including functional chemotaxis/oxidative and non-oxidative killing mechanisms [105]. 

A recently conducted study reported that the use of steroids, hyperglycemia, and COVID-19 infection may contribute to neutrophil dysfunction and endothelial damage during CAM. Avoiding the use of steroids in mild COVID-19 infections may prove to be helpful in reducing the risk of mucormycosis infection [106]. Further studies are required to confirm the precise relationship between COVID-19 infection, DM, and hyperglycemia, which may contribute to understanding the pathogenesis of CAM. The above-mentioned major host risk factors are described in Figure 2. 

### 3.5. Environmental Factors

Environmental risk factors can be a major cause of mucormycosis infection. Prolonged hospital stays, used face masks, unhygienic conditions, air-conditioning vents, and oxygen cylinders (with leaky humidifiers) can create a favorable environment for Mucorales growth and can further enhance the risk for contracting mucormycosis infection [41,107]. Hospital environments can be a major reservoir of *Mucorales* spp. due to inadequate hygienic practices. Identifying the source of Mucorales contamination is a major challenge, which leads to the delayed employment of preventive measures. Due to inadequate environmental-based studies in developing countries, the exact cause(s) of the higher risk of developing mucormycosis is not yet identified [108]. Recently, in a study conducted in 11 hospitals in India, samples were collected from the equipment and indoor/outdoor environment air. Although none of the hospital equipment was found to be contaminated with Mucorales, Mucorales species were isolated from patient’s used face masks and air-conditioning vents. Among isolated fungal species, the *Rhizopus* species was found to be the most frequently present species in a hospital environment [108]. 

During the SARS-CoV-2 pandemic, the use of industrial oxygen cylinders could be a probable risk factor for rising mucormycosis infections in COVID-19 patients. The industrial oxygen cylinders are different from medical oxygen cylinders. The use of industrial oxygen cylinders is associated with a risk of contamination and infection [107]. The use of tap and/or unfiltered water in oxygen cylinders can contribute to Mucorales growth in pipelines. Therefore, sterile and distilled water should be used in oxygen cylinders and humidifiers [109]. The exact cause of CAM emergence is unknown. There could be more contributing, unique risk factors of CAM, which remain to be identified. In a recent study, it was hypothesized that the burning of Mucorales-rich biomass, which includes cow dung and crop stubble, may result in the exposure of fungal spores to a large, human population. It may be considered as a major environmental risk factor for the CAM crisis in India [110].

### 3.6. Other Possible Risk Factors

A higher risk of CAM infection has been reported in people who had repeated (more than twice) nasopharyngeal swab testing for COVID-19 [106]. Self-medication without consulting a physician and the uncontrolled use of drugs may be linked to elevating the risk of mucormycosis infection. An *in vitro* study conducted by Muthu et al. reported that zinc-enriched media supported the better growth of *R. arrhizus* species, which indicates that the use of zinc supplements as immunity boosters could be a risk factor of mucormycosis infection [111]. Recently, Kumar et al. conducted a case–control study in an Indian rural area hospital and found an association between the self-medication of zinc and CAM. A significant number of about 89.1% of CAM cases had a history of zinc administration as immunity boosters, which indicated that zinc intake may be a risk factor contributing to the sudden rise in CAM cases [112]. 

In another study, Bilgic et al. hypothesized that the excessive use of antibiotics for the treatment of COVID-19 pneumonia may interfere with natural and protective microflora in the human body and make conditions favorable for Mucorales invasion [113]. Therefore, it is essential to address the problems, such as self-medication and excessive/uncontrolled use of drugs. There must be an awareness program for pharmacists and the general public regarding the side effects and risk of diseases associated with the uncontrolled use of drugs. Strict laws should be enforced by governments to control the extent of self-medication [114]. 

## 4. Pathophysiology and Immune Dysfunction in COVID-19-Associated Mucormycosis

Mucorales produce spores that are responsible for the initiation of infection. If a person inhales fungal spores through the nostrils from the atmosphere, then mucus and cilia help to clear the spores from the nasal cavity and windpipe. However, when spores gain entry into the sinus and pulmonary tract, they may germinate and proliferate inside the lungs when they find favorable conditions [23]. An *in vitro* study showed that Mucorales interact with TLR-2 (Toll-like receptor) on the cell surface of phagocytes, that lead to the activation of the downstream signaling pathway, which involves NF-κB activation and the release of various cytokines, such as IL-6 (interleukin) and TNF-α (tumor necrosis factor-alpha) [115]. In DM patients, due to the associated DKA, hyperglycemia, and corticosteroid therapy, the function of phagocytes became impaired, which diminished the host immune response against mucormycosis [116]. COVID-19 infection treated with certain drugs, such as immunosuppressants and corticosteroids, may decrease the ability of the immune system to fight against other pathogens [31,32]. Although corticosteroids are effective in the treatment of various respiratory illnesses, their excessive use may suppress the host immune system, resulting in an enhanced susceptibility to diseases, such as mucormycosis [117]. 

COVID-19 infection causes severe damage to lung tissue and the alveolar region, which provides an opportunity to other pathogens for causing infection. Furthermore, critically ill patients with severe COVID-19 pneumonia require mechanical ventilation, in which the proper flow of gases and airway pressure has to be maintained for individual patients. Improper mechanical ventilation can lead to lung injuries. Therefore, it is essential to understand the duration of symptoms, lung mechanics, and underlying pathophysiology to prevent lung injury during the mechanical ventilation of COVID-19 patients [118]. 

Fungal infections, such as mucormycosis, are mostly involved in causing damage to the pulmonary tract, so COVID-19 patients may be more prone to such infections [39,63]. In a recent study, it was reported that certain abnormalities, such as a reduction in B-, T- and NK-cell numbers and the premature activation of granulocytes in the nasopharyngeal region, were at their peak during the second week after the appearance of initial COVID-19 symptoms. Furthermore, the CAM cases were at their peak during the third week after the onset of initial COVID-19 symptoms. Although the emerging mutated variants of the coronavirus complicated COVID-19 disease management, the relationship between SARS-CoV-2 variants of concern and the development of CAM has not been explored much [106]. However, an association between the emergence of the B.1.617.2 (Delta) variant of SARS-CoV-2 and mucormycosis has been discussed. The majority of CAM cases were reported during the second wave of COVID-19 when the B.1.617.2 (Delta) variant was the predominant strain of SARS-CoV-2 prevalent in India [119]. Although there is a lack of evidence-based studies, the “oxygen crisis” during the second wave of COVID-19 in India may also be considered as an uncommon factor contributing to CAM pathogenesis [119]. In severe COVID-19 patients, the number of CD4+ T and CD8+ T lymphocytes decreases, which causes a condition known as lymphopenia [38]. This decrease in the number of immune cells may hamper the ability of the host defense response. It may dysregulate immune homeostasis, which may increase the susceptibility of COVID-19 patients to contract opportunistic fungal co-infections, such as mucormycosis [120]. COVID-19 infection downregulates the expression of spleen tyrosine kinases, involved in antifungal immune responses, which may contribute to invasive mucormycosis [121]. 

The mechanism of a platelet-mediated antifungal response may be due to the production of anti-inflammatory cytokines, such as TGF-β (transforming growth factor-beta), which activate other immune cells by interacting through their cell surface receptors, which further facilitate fungal clearance [122,123]. A decreased number of platelets is associated with a condition known as thrombocytopenia. Chang et al. reported mild thrombocytopenia in COVID-19 patients [124]. Since platelets are involved in antifungal immune responses, thrombocytopenia may increase the risk of mucormycosis infection. Furthermore, certain clinico-pathological features, such as corticosteroid- and DM-mediated immunosuppression, COVID-19-mediated cytokine storm, and endothelial damage, were found to be associated with the sudden rise in mucormycosis cases [125]. 

## 5. Interaction of Mucorales with Endothelial Cells

Mucorales spores may invade the endothelium of blood vessels by interacting with endothelial cell receptors through surface proteins. After invading the endothelial lining of blood vessels, Mucorales leads to the thrombosis of blood vessels by the occlusion of the blood supply, and ultimately causes severe tissue necrosis [25]. The interactions of Mucorales with endothelial cells and how it contributes to fungal pathogenesis and virulence has been studied by many researchers. Fungal spores enter the host’s tissue by breaching the mucosal membrane, and then interact with the extracellular matrix and find their way to the basement membrane where they bind to the protein laminin [126]. The receptor–ligand interaction occurs between the receptor, GRP78 present on the cell surface of endothelial cells and CotH3 ligand present on the cell surface of fungal spores. The binding of CotH3 with GRP78 facilitates fungal endocytosis [127]. 

GRP78 is a heat-shock protein that is produced by endothelial cells in stressed conditions. The ER (endoplasmic reticulum) of endothelial cells releases the GRP78 protein in response to stress [128]. ER stress may also be induced by the SARS-CoV-2 virus. This virus contains a spike protein, which stimulates the synthesis of the GRP78 protein. Studies reported that the serum of COVID-19 patients contained very high quantities of the GRP78 protein, about five times higher than normal controls. It has been reported that GRP78 also mediates SARS-CoV-2 invasion into host tissues or cells. In this way, COVID-19 infection may contribute to fungal internalization by upregulating GRP78 expression [129,130,131]. Recently, Chakrabarti et al. proposed that COVID-19 infection may provide a predisposition to Mucorales invasion by upregulating GRP-78 expression levels [121].

There are some other factors, such as the increased concentration of ketone bodies and blood glucose levels, which contribute to fungal invasion by enhancing GRP78 expression [25]. Studies conducted on a DKA murine model of mucormycosis found that during *Rhizopus oryzae* infection, the attenuation of CotH3 protein expression led to decreased tissue invasion and endothelium damage, indicating its possible role in the pathogenesis of mucormycosis [132]. 

## 6. Iron Metabolism and Mucor Growth

In humans, iron is present in a sequestered form with some proteins, such as ferritin and transferrin. This leads to the unavailability of free iron that is required by fungus for growth [133]. Various pathogens, such as Mucorales, are unable to utilize this sequestered form of iron, and due to this, their growth halts and they become dormant or removed by the body’s defense system. A recent study has shown the inability of *R. oryzae* to grow in iron-deficient media, which indicates the significance of iron in fungal growth and metabolism [134]. 

SARS-CoV-2 infection dysregulates iron homeostasis and increases the concentration of free iron in the blood. Usually, ferritin protects the body’s cells from free iron by storing it, but due to COVID-19-induced iron dysregulation, excessive free iron accumulates in the blood and sends signals to the liver to produce more ferritin [135,136]. Free iron may have some toxic effects on cells. Ferritin is an inflammation marker that acts as an indicator of SARS-CoV-2 pathogenesis. During COVID-19 infection, ferritin releases iron in the serum in response to acute inflammation. Cytokines, such as IL-6, and phagocyte activation also contribute to increasing the ferritin concentration and cause hyperferritinemia [137,138]. 

Bhadania et al. reported the extent of mucormycosis infection in COVID-19 patients by assessing serum ferritin levels. They reported that hyperferritinemia enhances the extent of Mucorales invasion. The serum ferritin levels were much higher in COVID-19 patients suffering from DM and hypertension [139]. Furthermore, elevated ferritin levels led to increased intracellular iron [140]. Intracellular free iron leads to the activation of reactive oxygen species within the cell, which produces free radicals; these free radicals cause the lipoperoxidation of the endothelial cell membrane. The damage to endothelial lining caused by ferritin and free iron-induced oxidative stress is responsible for the diffused inflammation of endothelial cells, which leads to endotheliitis [141]. The pathogenesis of CAM is described in Figure 1.

Iron transporters, such as ferroportin and hepcidin, play an important role in iron homeostasis [142]. The spike protein of the SARS-CoV-2 virus mimics hepcidin, which may dysregulate iron homeostasis and increase free-iron concentration inside the cell, which cause cellular damage [143,144]. Few *in vitro* studies found a direct relationship between the presence of iron and the growth of *R. oryzae* [145]. The virulence of Mucorales is dependent on the presence of elevated free iron in the blood [26]. The high-affinity iron permeases are a part of the reductase and copper oxidase complex, which are involved in iron uptake through the fungal cell surface [146]. The surface-reductase enzyme reduces ferric ions (less soluble) to ferrous ions (more soluble). Ferrous ions are captured by the copper oxidase–permease complex and then ferrous ions become available for fungal acquisition [147]. 

Another study reported that the FTR1 gene is associated with the overexpression of high-affinity iron permeases on the *R. oryzae* cell surface during murine infection. These high-affinity iron permeases are responsible for iron uptake in Mucorales, which leads to fungal growth and proliferation [148]. FTR1 overexpression may also contribute to iron uptake from blood hemoglobin. It has been reported that FTR1 helps in the internalization of the heme–Fe^2+^ complex. *R. oryzae* also expresses heme oxygenases, which aid in the intracellular degradation of the heme molecule and generation of free iron [148]. In this way, free iron may contribute to Mucorales pathogenesis. 

Furthermore, there could be a correlation between COVID-19 infection and high-free-iron concentration in serum, which contribute to CAM pathogenesis. In a recent study, a significant correlation was reported between CAM infection and elevated serum iron levels. Therefore, patients suffering from severe COVID-19 infection and with increased serum iron levels may be more susceptible to mucormycosis infection [149].

## 7. Current Diagnostics for COVID-19-Associated Mucormycosis

A direct microscopic examination is performed by staining specimens with H&E, methenamine silver, or an optical brightener (calcofluor white). The fragile nature of Mucorales makes this identification difficult; therefore, it should be performed carefully [150]. However, invasive fungal diseases, such as mucormycosis, may be diagnosed by a histopathological examination if characteristic features of the disease, such as angioinvasion and necrosis, are present in the tissue sample. The conventional diagnostic techniques lack sensitivity and specificity and may result in an incorrect diagnosis [151]. 

Computed tomography can be used to for the early detection of pulmonary mucormycosis in cancer patients [152]. Antibodies working against Mucorales are available commercially, which may be utilized in immunohistochemistry-based identifications [153]. Molecular tools may be used for diagnostic purposes in fresh biopsy tissue obtained from infected tissue. In immunocompromised patients, computed tomography and a galactomannan enzyme immunoassay can be used in lung biopsy tissue for the identification of invasive fungal infections [154]. 

PCR/ESI-MS (PCR–Electrospray Ionization Mass Spectrometry) is a good option for Mucorales hyphae identification within 6 h [155]. Formalin-fixed tissue, which is embedded in paraffin wax, may be used for identification, but as formalin may cause damage to DNA, fresh tissue is preferred for the precise identification of Mucorales [156]. Standardized PCR-based identification assays are promising for identification purposes. Various molecular targets, such as 18S and 25S ribosomal DNA, ITS (Internal Transcribed Spacer) region, and cytochrome b, may be used for the early diagnosis of Mucorales [157]. 

The limitations associated with the currently available diagnostics, such as high cost, non-specificity, lack of serological assays, and inability to identify novel species, make it challenging to diagnose mucormycosis at an early stage. The identification of fungal species at their species level is a prerequisite for a proper prognosis of infection [158,159].

## 8. Treatment Options for Mucormycosis

For the management of mucormycosis, the treatment options may be divided into different categories, which include first-line therapy, salvage therapy, adjunctive therapy, surgical treatment, and nutraceutical-based therapy. 

### 8.1. First-Line Therapy

First-line therapy involves high doses of liposomal amphotericin-B. Treatment with liposomal amphotericin-B should be initiated at an earlier stage of infection [160]. In a study involving 80 clinical isolates of Mucormycetes, amphotericin B was found to be the most potent antifungal drug, followed by posaconazole, itraconazole, and isavuconazole [161]. 

During the COVID-19 pandemic, a sudden increase in the demand for amphotericin B resulted in a shortage of the amphotericin-B drug [160]. Although the liposomal formulations of amphotericin B are less toxic, it is a highly expensive treatment option. Of note, antifungal drug-based treatment is not considered as effective for mucormycosis, due to its associated side effects and drug resistance [162,163]. 

### 8.2. Salvage Therapy 

In salvage therapy, posaconazole may be used, which acts effectively against Mucorales. Some studies reported better survival rates in mucormycosis patients after using posaconazole via oral-route (200 mg 4 times per day) treatment [164,165]. In an *in vitro* study, isavuconazole, a triazole drug, exhibited inhibitory effect against *Mucorales* spp. at a minimum inhibitory concentration: MIC: 1–4 mg/L [166]. It was reported that combination therapy using polyene and caspofungin at a dose of 5–10 mg/kg was found to be more effective than amphotericin-B monotherapy against rhino-orbital-cerebral mucormycosis [167]. In combinatorial therapy, amphotericin-B may be combined with either caspofungin or posaconazole [23]. 

Salvage therapy involving echinocandins, along with polyene, may be used if first-line therapy fails to control mucormycosis [150]. It was found that posaconazole at an MIC value in the range of 0.25–8 μg/mL could enhance the activity of amphotericin B against Mucor hyphae in an *in vitro* study [168]. Another study reported that calcineurin inhibitors, which include immunosuppressing agents, tacrolimus (MIC: 4 mg/L), cyclosporin A (MIC: 8 mg/L), and sirolimus (MIC: 4 mg/L), could enhance the *in vitro* antifungal activity of isavuconazole against *Mucor* species [169]. 

A separate study reported the use of L-AMB/isavuconazonium sulphate (MIC: 0.125–4.0 mg/L) combination therapy against experimental mucormycosis. L-AMB/isavuconazonium sulphate therapy was found to be effective against two *Mucorales* spp., namely, *Mucor circinelloides* and *Rhizopus delemar* [170]. Despite the availability of antifungal drugs, many middle-income countries cannot afford antifungal drugs due to the associated high cost of medication.

### 8.3. Adjunctive Therapy 

Adjunctive therapy is not recommended for the treatment of mucormycosis due to the lack of evidence-based studies. Some researchers conducted studies by using hyperbaric oxygen and Deferasirox against Mucorales. In the case of hyperbaric oxygen, survival rates were better in only DM patients, but other than diabetic patients, the survival rates were very low. It was reported that Deferasirox increased the mortality rates in mucormycosis patients [171,172]. Another study reported the use of statins against mucormycosis. Bellanger et al. reported that the administration of statins below a minimum inhibitory concentration could decrease the angioinvasion of *R. oryzae* and also induced its apoptosis [173].

It has already been discussed that iron helps in fungal growth and development. A recent study showed that lactoferrin, which acts as an iron chelator, may prove to be an adjunctive treatment option against mucormycosis [174]. Furthermore, anti-inflammatory drugs have been used, which inhibit the development of *Mucor* sporangia, and it has been reported that anti-inflammatory drugs selectively target the *Mucor* sporangia having high mitochondrial activity [175]. 

In a recently conducted study, Mrig et al. reported the use of a saturated solution of potassium iodide (SSKI) with liposomal amphotericin-B as an adjunctive therapy against *R. arrhizus*. The oral administration of SSKI in mucormycosis patients reduced the dose and duration of amphotericin-B treatment [176]. An *in silico* study evaluated the potential antifungal compounds obtained from marine sponges against CAM, and reported that antifungal compounds belonging to different classes, such as alkaloids, tetratomic glycosides, sesquiterpene phenols, and macrolides, could be used as promising therapeutic candidates against CAM [177]. As such, there is a lot of uncertainty surrounding the use of adjunctive therapy for the treatment of mucormycosis. 

### 8.4. Surgical Treatment

Since the treatment of mucormycosis is challenging due to poor drug penetration of necrotic tissues, surgical treatment is associated with better survival rates. In one study, surgical treatment lowered the risk of mortality in mucormycosis-infected patients [178]. In rhino-orbital-cerebral mucormycosis, the surgical removal of infected tissue was found to be effective [179]. ESCIMID, ECIL, and ECMM guidelines also recommend the surgical treatment of mucormycosis [156]. The surgical debridement of mucormycosis-infected tissue is required for the effective management of mucormycosis [163]. 

The Barrier draping technique may be used by surgeons to perform surgical operations on SARS-CoV-2-infected patients [179]. In a recent study, the wide surgical debridement of necrotizing tissue was performed for the imperative control of rhino-orbital mucormycosis [180]. One limitation is that surgery may be a costly treatment option, which may place a lot of burden on low-income settings.

### 8.5. Nutraceutical-Based Therapy

For the early management of COVID-19 infection, nutraceuticals may prove to be useful, which may further reduce the risk of CAM infection. Maurya et al. found that nutraceuticals, such as curcumin, zingiberene, theaflavin, berberine, resveratrol, and nimbin, showed a binding affinity to the ACE-2 receptor, which may impair the binding of the SARS-CoV-2 virus to the surface of host cells [181]. 

Another study evaluated the potential of curcumin (800 μg/mL) for the *in vitro* inhibition of *R. oryzae* [182]. It was reported that essential oils obtained from the rhizome of *Boesenbergia pandurata* (fingerroot) presented antifungal properties. It was found to be effective against Mucor species at an MIC of 0.63 g/L [183]. Novel treatment options that prevent a COVID-19-associated cytokine storm are strongly required. In this regard, the anti-inflammatory properties of spices may decrease cytokine storms in COVID-19 patients [184]. Nutraceuticals may also reduce the risk of COVID-19-associated infections, such as mucormycosis [185]. 

In a recent *in silico* study, a phytocompound, namely, quercetin obtained from *Azadirachta indica* and *Curcuma longa*, was found to be a promising therapeutic candidate against CAM infection [186]. Although the use of nutraceutical-based therapy is not yet much explored, more *in vivo* studies may be required to strengthen the *in vitro* research findings. Various treatment options against CAM are also described in Figure 2.

## 9. Guidelines for the Management of COVID-19-Associated Mucormycosis

The Directorate General of Health Services (DGHS) has provided guidelines for the management of CAM in India. Mucormycosis treatment should be initiated at an early/initial stage to reduce mortality rates. Treatment options include a combination of antifungal therapy and surgical debridement of infected tissue, although amphotericin B (1–1.5 mg/kg) may be used if liposomal formulations of amphotericin B are not available. However, the treatment of choice is liposomal formulations of amphotericin B at a dose of 5 mg/kg. Furthermore, in the case of CNS involvement, a dose of 10 mg/kg of body weight can be used. The amphotericin-B dose should be diluted in 5–10% of dextrose. Treatment using amphotericin B should be continued until a positive response is observed in terms of disease stabilization/resolution. Treatment monitoring may take several weeks. Following amphotericin-B therapy, salvage therapy with oral posaconazole (300 mg/day) or isavuconazole (200 mg/day) can be initiated. Antifungal therapy should be continued until a resolution of mucormycosis symptoms and disappearance of radiological signs of mucormycosis are observed [187]. The potential of isavuconazole or posaconazole as predominant forms of antifungal therapy for CAM was evaluated in a recent retrospective observational case study. It was reported that isavuconazole or posaconazole could be employed as sole antifungal therapy for CAM without using first-line therapy with amphotericin B [188]. 

## 10. Potential of Immunotherapies against COVID-19-Associated Mucormycosis

In immunocompromised hosts, the immune system becomes less functional, which may further enhance host susceptibility to fungal infections. Mucor species are highly pathogenic fungal species causing necrotizing lung infection and, in many cases, can affect host brain tissue. There are limited therapeutics for invasive fungal infections [189]. Mucormycosis requires an early diagnosis and treatment. Previous studies have shown that crude extracts obtained from fungal species may have antigenic properties. A total of eleven fungal extracts were tested and characterized by SDS-PAGE and immunoblot analysis. It was found that most of the fungi shared antigenic proteins [190]. A monoclonal IgG antibody was raised against the extracellular carbohydrate moieties of *Mucor racemosus*. It was reported that such a monoclonal antibody could be used in the detection of pathogenic Mucor species in humans [191]. In another study, a monoclonal antibody (2DA6) was produced, which recognized α-1, 6 mannan, an antigen found in fungi, including *Rhizopus*, *Mucor*, *Fusarium*, *Candida*, and *Aspergillus*. It was reported that the detection of such conserved antigens using immunoassays could aid in the diagnosis of a broad range of fungi [192]. 

The identification of fungal antigenic components may prove to be beneficial in the early diagnosis of mucormycosis infection. A monoclonal antibody, MUC5B, was used in the immunohistochemistry-based detection of fungal pathogens. It was reported that the MUC5B antibody could be used to distinguish between *Aspergillus* and *Mucor* species [193]. Since high-risk patients, which include immunocompromised patients, having underlying diseases are more prone to develop mucormycosis, there is a need for effective antifungal therapies to control the fungal infection [194]. 

In recent decades, no significant improvement has been observed in the prognosis of mucormycosis, due to the challenges associated with its early diagnosis and the limited availability of antifungal agents. The hematopoietic stem cell transplant is associated with a high risk of mucormycosis, accounting for almost 90% of mortalities. Schmidt et al. reported that the reconstitution of host immunity following a hematopoietic stem cell transplant may be essential to combat fungal infections. They demonstrated that IL-2 pre-stimulated NK cells could kill mucormycetes, and the early administration of human NK cells was found to be more effective in killing mucormycetes [195]. 

Although few studies that support the use of combinatorial immunotherapies have been conducted, further research is required in this area. One study demonstrated the feasibility of using combinatorial immunotherapy against mucormycosis. They treated three children having invasive fungal infections and hematological malignancies with combination antifungal therapy along with granulocyte colony-stimulating factor (G-CSF)-mobilized granulocyte transfusions. The immunotherapeutic approaches effectively improved the survival rate of high-risk patients [196]. 

Treatment monitoring can be a used as a tool to monitor the host’s response to antifungal treatment. Cytokines act as immunological biomarkers, which aids in the diagnosis as well as treatment monitoring of invasive fungal infection. A cytokine-based diagnosis of invasive fungal infection is cost effective and rapid, which may result in the early and effective management of mucormycosis [197]. A separate study demonstrated the role of IFN-γ in conferring partial protection against pulmonary mucormycosis in a mice model of experimental mucormycosis [198]. 

In another recent study, the anti-CotH3 antibody was found to be protective in a murine model of mucormycosis. The mechanism of protection was reported as antibody-mediated opsonophagocytic activity against *R. delemar*. Furthermore, the anti-CotH3 monoclonal antibody exerted a synergistic effect with antifungal drugs against *R. delemar* spp. This study indicated the role of anti-CotH3 antibodies in adjunctive/combination/antibody-mediated antifungal therapies [199]. Additionally, *R. delemar* spores contain the CotH7 antigen, which is involved in the invasion of alveolar epithelial cells via an interaction with β1-integrin receptors. Alqarihi et al. reported that anti-β1 integrin antibodies bind to β1-integrin receptors and inhibit the invasion of *R. delemar* through alveolar epithelial cells, and conferred protection in a mice model of pulmonary mucormycosis [200]. 

For long-term protection against mucormycosis infection, vaccines are urgently required. In this regard, Areitio et al. identified immunoreactive protein antigens from *M. circinelloides* as vaccine candidates. Fungal protein antigens, such as enolase, triosephosphate isomerase, and heat-shock protein HSS1, were tested in a mice model of mucormycosis. It has been reported that these protein antigens may prove to be useful for future vaccine development against mucormycosis infection [201]. 

Another study conducted by Ibrahim et al. reported that several vaccine compositions, including the FTR (high-affinity iron permease) polypeptide, antibody inhibitor of FTR, and small interfering RNA, could confer protection against *R. oryzae*-mediated mucormycosis in a mice model of DKA [202]. In another study, using an immunoinformatic approach, Araf et al. designed a multivalent peptide vaccine against four different virulent *Mucorales* spp. (*R. oryzae*, *R. stolonifer*, *R. azygosporus*, and *M. circinelloides*) targeting the FTR1 protein. A detailed *in silico* analysis has shown that this anti-fungal vaccine could prove to be an effective therapeutic agent against CAM infection [203]. 

More recently, Pritam et al. designed two multi-epitope vaccine candidates against *R. delemar* using immunoinformatics and reverse vaccinology approaches. In brief, they selected four genome-derived predicted antigens and four experimentally reported antigens, which were then subjected to B- and T-cell epitope predictions. They reported that both vaccine candidates could induce innate and adaptive immune responses against *R. delemar* [204]. In another *in silico* study, Naveed et al. designed a vaccine construct against CAM infection, targeting GRP78 and TLR2 proteins from two *Mucorales* spp., namely, *R. oryzae* and *M. circinelloides* [205]. Further *in vitro* and *in vivo* pre-clinical studies are required to evaluate the efficacy and safety of such vaccine constructs. Although few studies are available, more evidence-based studies are required to assess the potential of immunotherapy against mucormycosis. Owing to its specificity and effectiveness, this area of immunotherapeutics needs to be explored in the future. 

## 11. Conclusions

Although various population-based studies were conducted in Western countries, which helped in the estimation of mucormycosis incidence, due to the lack of population-based studies and insufficient number of sophisticated laboratories for fungal diagnosis in developing countries, it has been very difficult to estimate the exact incidence of mucormycosis. Additionally, there is a considerable difference between risk factors that contribute to mucormycosis infection in developed and developing countries. For instance, in developing countries, COVID-19 infection, along with the presence of various co-morbidities, such as chronic renal failure, orbital infarction syndrome, post-tuberculosis, long-term ICU stays, self-medication, used masks, use of industrial oxygen cylinders, oxygen cylinders with leaky humidifiers, and air-conditioning vents, represent major risk factors that may lead to alarmingly high rates of mucormycosis infection. On the other hand, patients with COVID-19-induced immunosuppression; hematological malignancies, such as neutropenia; and organ transplant recipients represent the main risk factors that may be responsible for mucormycosis incidence in developed countries. Notably, corticosteroid therapy, DM, DKA, and hyperglycemia are common and major risk factors of mucormycosis among COVID-19 patients in both developed and developing countries. The uncontrolled use of steroids and self-medication must be discontinued to tackle rising incidences of CAM. Moreover, the maintenance of low blood glucose levels in diabetic patients can prove to be a key approach for controlling hyperglycemia. The use of zinc supplements should be initiated only after a consultation with a physician to minimize the probable risk of mucormycosis. Since fungal culture-based identification assays usually produce incorrect results, and diagnostics for *Mucorales* spp. are performed using expensive and high-end or advanced techniques, such as MALDI-TOF, which are not feasible for middle-income countries, there is an urgent need for the development of cost-effective diagnostics for the precise identification of patients suffering from mucormycosis. Further studies need to be conducted for the identification of early biomarkers of mucormycosis. To date, surgery on infected tissue followed by antifungal therapy is the only recommended treatment option. Critical research gaps still exist, regarding the treatment of mucormycosis, including the use of salvage therapy, adjunctive therapy, combination antifungal drug-based therapy, and the evaluation of treatment responses in patients. The development of animal-based experimental models of mucormycosis for conducting pre-clinical studies may fill these research gaps. Furthermore, little is known about the use of nutraceutical-based therapy and immunotherapy against mucormycosis. Further research is required to resolve the global threat associated with CAM infection amid the SARS-CoV-2 pandemic.

## 12. Current Research Gaps

- Non-reporting of many cases leading to incomplete incidence rates.
- Lack of awareness leading to delay in seeking healthcare.
- Lack of knowledge among physicians.
- Financial constraints restricting the use of surgeries/other costly options in LLMICS.
- Use of improper antifungals and steroids in CAM.
- Research on overcoming drug resistance and improving the efficacy of antifungal drugs.
- Lack of environmental assessment studies for the identification of *Mucor* spp. sources.
- Identification of clinical biomarkers for assessing the risk of mucormycosis in high-risk populations and to enable early antifungal prophylaxis and therapy.
- Lack of research for developing rapid and cost-effective methods for early diagnosis.
- Lack of research for developing specific therapeutic strategies.
- Novel immunotherapies, such as antibody therapy; immunomodulators; adjunctive therapy, including cytokines; and combination therapy, including drugs and antibodies; are needed urgently.
- Need for vaccine development, which can prevent mucormycosis.
- Lack of studies on the genetic aspects of mucormycosis for understanding virulence.
- Need for more *in vivo* studies for studying various aspects of CAM.
- Development of animal models for mucormycosis.

## 13. Future Directions

To date, there are no standard protocols that can be implemented to treat mucormycosis in COVID-19 patients. Therefore, there is an overwhelming need to formulate a standard protocol for treating mucormycosis infections in COVID-19 patients. In addition to the development of new treatment options, there is an emergent need for developing rapid, cost-effective, and specific methods to enable the early diagnosis of COVID-19-associated mucormycosis. Novel immunotherapies that can be used in immunosuppressive conditions are required, using cytokines and/or antibodies. The research should also focus on deciphering the mechanisms that confer resistance to various antifungal drugs. Furthermore, it is necessary to perform and validate novel treatment options in all fungal species causing mucormycosis infections. In light of these associated challenges, it is essential that large-scale clinical trials are designed and conducted in mucormycosis patients globally, in order to identify all the associated factors between COVID-19 and mucormycosis and the ways to control them, which can result in reduced morbidity and mortality rates.

## Figures and Tables

**Table 1:** Summary of various case reports of COVID-19-associated mucormycosis discussed in this article. DM, diabetes mellitus and DKA, diabetic ketoacidosis. EAT, empiric antifungal therapy or observation-based antifungal therapy is followed in high-risk patients who have persistent fever after 4–7 days of broad-spectrum anti-bacterial drugs and no identified infection source. Some of the drugs used in EAT are amphotericin B, caspofungin, micafungin, voriconazole, itraconazole, and fluconazole.

S.N	No. of Cases	Gender/Age	*Mucorales* spp.	Location of Mucormycosis	Risk Factors	COVID-19 Diagnosis	Treatment	Patient’s Outcome	Ref.
1	One	F/24	*Lichtheimia*	Rhino-orbital	DM and DKA	RT-PCR	Amphotericin B	Died	[46]
2	One	M/79	*Rhizopus arrhizu*s	Pulmonary	DM	PCR	Amphotericin B		[47]
3	One	M/59	*Rhizopus microsporus*	Pulmonary	Neutropenia	RT-PCR	-	Died	[48]
4	Four	M/>50	*R. microsporus*, *Lichtheimia ramosa*, *R. arrhizus*	Rhino-orbital-cerebral, pulmonary, and disseminated	DM And obesity reported in two patients	RT-PCR	EAT	3 Died, 1 Lived	[49]
5	Fifteen	M/F/14–71	-	Rhino-orbital	DM and corticosteroid therapy	RT-PCR	Combined antifungal therapy and orbital exenteration	7 Died, 8 Lived	[50]
6	One	F/61	-	Rhino-orbital	Corticosteroid therapy	PCR	Surgery	Lived	[51]
7	Two	M/F/40	-	Rhino-orbito-cerebral and rhino-orbital	Corticosteroid therapy	PCR, CT scan	Amphotericin B and surgery	1 Died, 1 Lived	[52]
8	One	F/73	*Mucor*	Paranasal and orbital	DM and chronic renal disease	PCR	Amphotericin B	Died	[53]
9	One	M/60	*Rhizopus*	Rhino-orbital	DM and hyperglycemia	-	Amphotericin B and posaconazole	Died	[54]
10	One	M/41	-	Rhino-cerebral	DM and DKA	RT-PCR	Amphotericin B and surgery	Lived	[55]
11	Two	M/>36	-	Rhino-orbital-cerebral	DM, DKA, and corticosteroid therapy	-	EAT	Both Died	[56]
12	One	M/44	-	Rhino-orbital	-	RT-PCR	Surgery and antifungal therapy	Died	[57]
13	Two	M/F/>60	-	Rhino-orbito-cerebral	DM and steroid therapy	RT-PCR	Amphotericin B	1 Died, 1 Lived	[58]
14	One	M/20	-	Rhino-orbital-cerebral	DM and corticosteroid therapy	-	Amphotericin B and surgery	Died	[59]
15	One	F/65	-	Rhino-cerebral	DM and corticosteroid therapy	-	Antifungal therapy and insulin	Lived	[60]
16	One	M/61	-	Rhino-orbital	DM and corticosteroid therapy	RT-PCR	Amphotericin B and isavuconazole	-	[61]
17	One	F/>50	*Rhizopus* spp.	Rhino-orbital-cerebral	DM and hyperglycemia	RT-PCR	Amphotericin B	Lived	[62]
18	Ten	M/F/>53	*Rhizopus* spp.	Rhino-orbital	DM and corticosteroid therapy	RT-PCR	EAT	1 Died, 9 Lived	[63]
19	Eighteen	M/F/35–73	-	Rhino-cerebro-orbital	DM and corticosteroid therapy	RT-PCR	Orbital exenteration	6 Died, 11 Lived, 1 Lost to follow-up	[64]
20	One	M/60	-	Rhino-orbital	DM and corticosteroid therapy	RT-PCR	EAT	Died	[65]
21	One	M/28	-	Rhino-orbital	HIV and hypocomplementemia	RT-PCR	Amphotericin B and surgery	Lived	[66]
22	Ten	M/F/23–67	*Rhizopus* and *Mucor* spp.	Orbital	DM and DKA	RT-PCR	Antifungal drug therapy and surgery	4 Died, 6 Lived	[67]
23	One	M/66	*Rhizopus* spp.	Pulmonary	Arterial hypertension	-	EAT,	Died	[68]
24	One	M/38	*Rhizopus oryzae*	Sino-orbital	-	RT-PCR	Fluconazole, amphotericin B, surgery	Lived	[69]
25	One	M/66	Mucor	Rhino-orbital	DM and corticosteroid therapy	-	Amphotericin B and surgery	Lived	[70]
26	One	F/32	-	Paranasal	DM	CBNAAT	Amphotericin B	Lived	[71]
27	One	M/27	Mucor	Pulmonary	-	-	Amphotericin B	Died	[72]
28	One	M/39		Mandibular	-	-	Posaconazole	Lived	[73]
29	Two	M/F/11–13	*Rhizopus arrhizus*	Rhino-orbital-cerebral	DM and hyperglycemia	COVID-19 antibody test	EAT and surgery	Both Lived	[74]
30	One	M/48	-	Pulmonary and sino-nasal	DM and DKA	-	Antifungal therapy and surgery	Died	[75]

## Data Availability

Not applicable.
